# Using the multiphase optimization strategy (MOST) to optimize an HIV care continuum intervention for vulnerable populations: a study protocol

**DOI:** 10.1186/s12889-017-4279-7

**Published:** 2017-05-04

**Authors:** Marya Viorst Gwadz, Linda M. Collins, Charles M. Cleland, Noelle R. Leonard, Leo Wilton, Monica Gandhi, R. Scott Braithwaite, David C. Perlman, Alexandra Kutnick, Amanda S. Ritchie

**Affiliations:** 1 Center for Drug Use and HIV Research, Rory Meyers College of Nursing, New York University, New York, NY USA; 2 The Methodology Center and Department of Human Development and Family Studies, Pennsylvania State University, State College, Pennsylvania, PA USA; 3 Department of Human Development, State University of New York at Binghamton, Binghamton, NY USA; 4 Division of HIV, Infectious Diseases, and Global Medicine, School of Medicine, University of California San Francisco, San Francisco, CA USA; 5 Department of Population Health, New York University School of Medicine, New York, NY USA; 6 Department of Infectious Diseases, Mount Sinai Beth Israel, New York, NY USA; 7 Faculty of Humanities, University of Johannesburg, Johannesburg, South Africa

**Keywords:** HIV care continuum, Antiretroviral initiation, HIV care, Multiphase optimization strategy, MOST, African American, Black, Hispanic, Disparities, Intervention

## Abstract

**Background:**

More than half of persons living with HIV (PLWH) in the United States are insufficiently engaged in HIV primary care and not taking antiretroviral therapy (ART), mainly African Americans/Blacks and Hispanics. In the proposed project, a potent and innovative research methodology, the multiphase optimization strategy (MOST), will be employed to develop a highly efficacious, efficient, scalable, and cost-effective intervention to increase engagement along the HIV care continuum. Whereas randomized controlled trials are valuable for evaluating the efficacy of multi-component interventions *as a package*, they are not designed to evaluate *which specific components* contribute to efficacy. MOST, a pioneering, engineering-inspired framework, addresses this problem through highly efficient randomized experimentation to assess the performance of individual intervention components and their interactions. We propose to use MOST to engineer an intervention to increase engagement along the HIV care continuum for African American/Black and Hispanic PLWH not well engaged in care and not taking ART. Further, the intervention will be optimized for cost-effectiveness. A similar set of multi-level factors impede both HIV care and ART initiation for African American/Black and Hispanic PLWH, primary among them individual- (e.g., substance use, distrust, fear), social- (e.g., stigma), and structural-level barriers (e.g., difficulties accessing ancillary services). Guided by a multi-level social cognitive theory, and using the motivational interviewing approach, the study will evaluate five distinct culturally based intervention components (i.e., counseling sessions, pre-adherence preparation, support groups, peer mentorship, and patient navigation), each designed to address a specific barrier to HIV care and ART initiation. These components are well-grounded in the empirical literature and were found acceptable, feasible, and promising with respect to efficacy in a preliminary study.

**Methods/design:**

Study aims are: 1) using a highly efficient fractional factorial experimental design, identify which of five intervention components contribute meaningfully to improvement in HIV viral suppression, and secondary outcomes of ART adherence and engagement in HIV primary care; 2) identify mediators and moderators of intervention component efficacy; and 3) using a mathematical modeling approach, build the most cost-effective and efficient intervention package from the efficacious components. A heterogeneous sample of African American/Black and Hispanic PLWH (with respect to age, substance use, and sexual minority status) will be recruited with a proven hybrid sampling method using targeted sampling in community settings and peer recruitment (*N* = 512).

**Discussion:**

This is the first study to apply the MOST framework in the field of HIV prevention and treatment. This innovative study will produce a culturally based HIV care continuum intervention for the nation’s most vulnerable PLWH, optimized for cost-effectiveness, and with exceptional levels of efficacy, efficiency, and scalability.

**Trial registration:**

ClinicalTrials.gov, NCT02801747, Registered June 8, 2016.

## Background

Even with recent important advances in the efficacy and tolerability of HIV treatment [1–6], serious gaps persist in the HIV care continuum in the United States [7, 8]. The Centers for Disease Control and Prevention estimates that of 1.2 million Americans living with HIV, 60% are not retained in HIV care; 63% are not taking antiretroviral therapy (ART); and 70% have detectable HIV viral load (VL) [9]. Poor engagement along the HIV care continuum increases risk for morbidity and early mortality [10–12], hospitalizations and increased health care costs [13, 14], and risk of forward transmission of HIV. Indeed, poor retention in HIV primary care is a principal cause of HIV/AIDS-related mortality [15–18], and lack of ART initiation further places persons living with HIV (PLWH) at elevated risk for substandard CD4 and VL outcomes [11, 19, 20].

Because most PLWH are African American/Black or Hispanic [21], gaps in engagement along the HIV care continuum are concentrated among these populations. Moreover, compared to their White peers, African American/Black and Hispanic PLWH (AABH-PLWH) are more likely to be diagnosed late in the course of their HIV disease, delay uptake of ART, discontinue ART, and to have higher rates of morbidity and earlier mortality from HIV [22–25]. Further, these racial/ethnic disparities are found among all major risk categories; namely, persons who inject drugs (PWID), men who have sex with men (MSM), and heterosexuals [26, 27]. The Centers for Disease Control and Prevention, Office of AIDS Research [28], and National HIV/AIDS Strategy [29] have stressed the importance of eliminating racial/ethnic disparities in HIV health outcomes, thereby signaling the need for culturally based HIV care continuum interventions [29–31].

### The MOST framework

The primary goal of the present study is to use the innovative multiphase optimization strategy (MOST) to select *individual intervention components* to comprise an optimized behavioral intervention, where the optimized intervention is the one that provides the greatest improvement in health outcomes achievable within the specified resource constraints [32]. MOST is an engineering-inspired framework and systematic method for identifying the optimized combination of intervention components *before* testing an intervention in a resource-intensive randomized controlled trial (RCT). MOST consists of three stages: 1) preparation, 2) optimization, and 3) evaluation of the optimized intervention in an RCT [32]. While the RCT is an excellent approach for evaluation of an intervention package as a whole, it was never intended to provide information about the performance of the *individual components* making up the intervention package. By contrast, MOST calls for empirically examining the efficacy of each separate intervention component, along with its resource requirements and costs.

### Objectives of the present study

In the present study, the goal is to select the set of intervention components likely to improve health outcomes to the greatest extent per dollar spent, yielding a cost-effective, efficient, and scalable culturally based behavioral intervention for AABH-PLWH. In recent preliminary research, we identified a set of promising intervention components for AABH-PLWH not taking ART and poorly engaged in HIV care [33, 34]. In the present study, an innovative and economical fractional factorial experimental design will be used to examine the effects of a set of five individual intervention components, their interactions, as well as mediation and moderation effects for each individual intervention component, providing a *detailed look* at the mechanisms by which each component works. Then, in the optimization process, based on modeling analyses, we will identify the combination of intervention components (likely 2–3 components) with the greatest levels of efficacy and cost-effectiveness, eliminating poorly performing, costly, or ineffective components. This new combination of components is called the “optimized intervention” [35–37]. The optimized intervention developed using this powerful new approach has the potential to make a major impact on engagement in HIV care and uptake of ART among AABH-PLWH, improving the health of this population, reducing forward transmission of HIV, and decreasing racial/ethnic HIV disparities – all national priorities [28, 29, 38, 39]. This project will be the first application of the MOST framework in the field of HIV prevention and treatment, and will result in the first optimized intervention aimed at improving engagement along the HIV continuum of care using biological outcomes (namely, CD4 and VL levels).

### Aims of the study

Thus the aims of the present study are:

Aim 1: Using a highly efficient experimental design, identify which of five intervention components contribute meaningfully to improvement in the primary outcome, HIV viral suppression, and secondary outcomes, absolute HIV viral load, ART adherence, and engagement in HIV primary care, all assessed via objective biomarkers or through the medical record.

Aim 2: Identify mediators and moderators of the efficacy of each intervention component (e.g., substance use history, sexual minority status), and also of interaction effects between components.

Aim 3: Using a mathematical modeling approach, build the most cost-effective and efficient intervention package from the components found to be efficacious in Aim 1.

## Methods/design

### Overview of the study

**Fig. 1:**
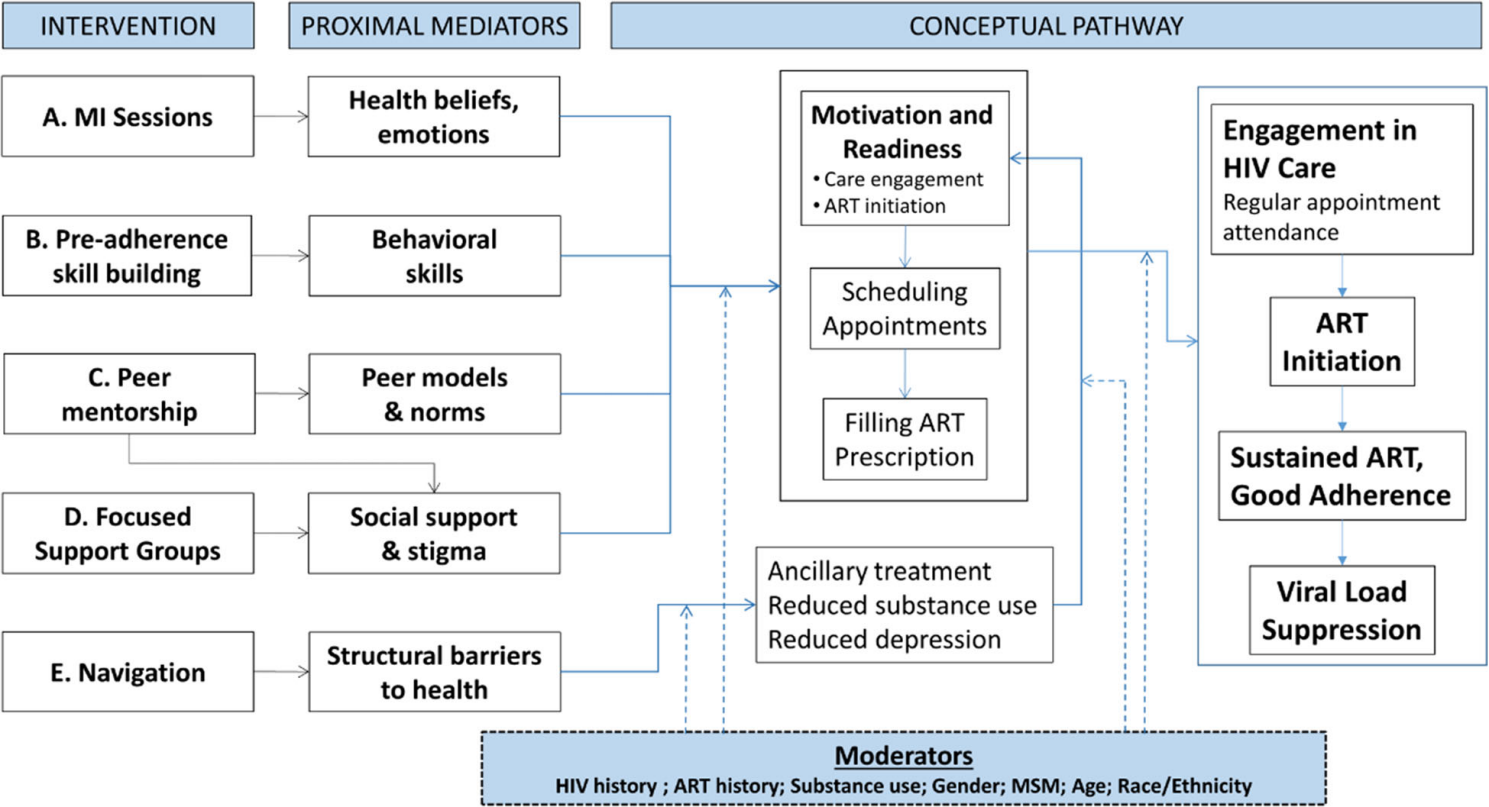
Conceptual model grounded in the theory of triadic influence and self determination theory

The present study focuses on African American/Black and Hispanic PLWH not well engaged in HIV care nor taking ART, referred to as “PLWH-NECTA”. We will enroll a heterogeneous sample of PLWH-NECTA (with respect to age, substance use, mental health, and sexual minority status). PLWH-NECTA are not typically found in HIV clinics. Instead, participants (*N* = 512) will be recruited with a proven hybrid sampling method using targeted sampling and peer recruitment, described below [33]. The present study is comprised of three stages: (1) Refinement (6 months); (2) Implementation, Cost Effectiveness Analysis, and Optimization (48 months); and (3) Final (6 months). Intervention optimization in stage 2 will proceed as follows: Five promising individual intervention components, grounded in an integrated social-cognitive theory (the theory of triadic influence combined with self-determination theory), will be examined by means of a fractional factorial experiment. The five intervention components, each of which is guided by the motivational interviewing counseling approach, and described in detail below, are: (A) Motivational Interviewing (MI) individual counseling sessions; (B) Pre-adherence preparation; (C) Peer mentorship; (D) Focused support groups; and (E) Navigation. Each component addresses one theoretical mediator or one small set of theoretical mediator(s) linked to known barriers to good engagement in HIV care and ART uptake among PLWH-NECTA, as shown in the study’s conceptual model (Fig. 1), and described below. All participants will receive a Core intervention session and be randomly assigned to one of 16 experimental conditions. Time and cost expenditure data for each intervention component will be collected. Then, mathematical modeling based on the results of the experiment will determine the most efficacious and cost-effective combination of intervention components, eliminating ineffective components.

### Theoretical model

The present study is guided by a theoretical model incorporating the theory of triadic influence [40] and self determination theory [41, 42]. The theory of triadic influence is a multi-level social-cognitive theory articulating three “streams of influence” acting simultaneously on health behavior; namely, the individual, social, and structural. Complementing the theory of triadic influence, self determination theory highlights the importance of durable, high-quality, intrinsic motivation for behavior change [41, 42]. The integrated theoretical model assumes the lack of HIV care and ART initiation are not independent: those who fear or otherwise decline ART present less frequently for HIV care [43, 44], and those not well engaged in HIV care rarely gain access to ART [21]. Importantly, these two gaps in the HIV care continuum – poor engagement in HIV care and low uptake of ART - are largely driven by the same set of multi-level risk factors and barriers [33, 34]. Guided by this integrated theoretical model, we next describe the primary barriers AABH-PLWH experience to both HIV care and ART initiation with sustained good adherence [45–47].

### Description of barriers to HIV care and ART

At the individual level of influence primary barriers to HIV care/ART for AABH-PLWH include negative health beliefs such as medical distrust, negative outcome expectancies, low levels of “readiness” [48–52], and negative emotions about care/ART, including fear [53–55]. Indeed, the primacy of fear as a barrier; namely, fear of being pressured to take ART in health care settings, of ART’s side effects and toxicities, and possible negative effects on relationships if on ART, cannot be over-stated [56, 57]. Substance use is another common barrier [33, 58–61], as are mental health concerns, primarily depression [62–65]. Further, lack of knowledge about care/ART guidelines [48, 66, 67] impedes ART/care, and PLWH often decline ART because they lack behavioral skills to maintain adherence to ART [68, 69].

Barriers at the social level of influence include a lack of positive “successful” peer role models who are regularly engaged in HIV care and taking ART with good adherence, who can challenge prevalent social/peer norms that health care systems cannot be trusted and ART is toxic and should be avoided [43, 44, 46]. Social isolation and low levels of social support also impede HIV care and ART use [70, 71], as does HIV stigma, compounded by stigma associated with poverty, substance use, and/or sexual minority status [72–74].

At the structural level of influence, barriers include challenges negotiating the health care system, including relations with providers [75, 76], transportation problems, and access to care for substance use and mental health concerns, as well as HIV [44, 48, 77]. Interventions may not eliminate structural barriers, but can reduce their effects by increasing participants’ options [78]. Barriers at all three levels are commonly rooted in poverty [44, 77, 79, 80] and combine synergistically to reduce AABH-PLWH’s motivation, behavioral skills, and access to HIV care and ART. On the other hand, factors facilitating good health outcomes operate concurrently with barriers, including intrinsic motivation to achieve good health [44, 81–84] and supportive network members [85]. As shown in Fig. 1, and described in more detail below, the present study will test a set of intervention components designed to address the primary barriers AABH-PLWH experience to HIV care and ART initiation at these three levels of influence.

### The present study attends to the needs of MSM

African American/Black and Hispanic MSM are greatly over-represented among the population of PLWH, making up more than half of the population of PLWH nationally [86]. Similar to other subgroups of AABH-PLWH, African American/Black and Hispanic MSM have suboptimal rates of linkage to care, retention in care, ART initiation, and HIV viral suppression [30]. Prior epidemiologic research highlights a number of clinical and socio-structural factors that create barriers to engagement along the HIV care continuum for African American/Black and Hispanic MSM. These include stigma related to HIV, as well as to sexual minority status, substance use, stress, and depression [72, 87–90]. The present study includes a focus on this critical subpopulation of PLWH. We estimate 55–60% of males in the present study will be MSM [33, 34].

### The present study addresses substance use and mental health concerns

Drug and alcohol use, and substance use problems, are endemic among PLWH [59, 60] and serve as major barriers to engagement along the HIV care continuum [59–61, 91, 92]. Cocaine, marijuana, opioids, and alcohol are the most frequently used substances, and poly-substance use is common [59, 92]. While *recent* injection drug use is not highly prevalent in this population (<4%) [60, 92], lifetime injection drug use prevalence is substantial (~17%) and associated with poor HIV outcomes [62], including delayed HIV diagnosis, reduced entry into and retention in HIV care, delayed initiation of ART, inferior adherence to ART [93, 94], and poor treatment outcomes [59]. Yet substance use does not preclude engagement in HIV care and good ART adherence [95], and substance use problems, while they may be serious, are addressable. Among PWID, opioid substitution therapy is associated with better adherence to ART [95–98], and a number of promising behavioral interventions have been developed for substance users living with HIV [97, 99–101]. Given the critical role substance use plays in HIV disparities, intervention efforts for HIV-infected substance users are vital [102]. Based on our own research [33, 74] and on national data [92], we estimate 55% of participants in the present study will be current substance users, primarily non-injectors, 25% will be past users (including PWID), and 20% will be non-users. Relatedly, mental health problems are widespread among AABH-PLWH, mainly depression and anxiety. We estimate 60–65% of the sample in the present study will evidence mental health distress at clinically significant levels [33, 34].

### Explanation for the choice of intervention components to be tested

The intervention components to be evaluated in the present study were developed and tested as a packaged intervention in a previous intervention development RCT. The intervention, called “Heart to Heart” (HTH), was highly efficacious, producing substantial reductions in VL, the study’s primary outcome, assessed via the medical record. Further, the intervention was highly acceptable and feasible, including for substance users, sexual minorities, and both males and females, and retention was excellent (> 95% attended the intervention; 90% completed a 4-month follow up assessment and 80% complete the 8-month follow up assessment) [34]. Rates of ART initiation were similar across arms (~ 58%) but 8 months post-baseline, participants in the intervention arm were three times more likely to evidence “good” (that is, 7 day/week) adherence (60% vs. 26.7%; *p* = 0.087; OR = 3.95), as assessed via ART concentrations in hair samples [103], and had significantly lower VL (intervention log_10_ VL = 1.63 [SD = 0.67], controls 2.51 [SD = 1.55], OR = 3.70; *p* = 0.02) than controls based on medical records.

Findings from the HTH study as well as the larger empirical literature on interventions for PLWH formed the basis for the selection of individual intervention components to be tested in the present study. We used the following guidelines for selecting components. Each component must: address one or one small set of theoretical mediator(s); be distinct from the others in content, length, delivery method, and/or approach; have, at minimum, preliminary evidence of efficacy or promise in the empirical literature; have been found feasible for and acceptable to the population under study; not require that any other component be administered along with it in order to be efficacious; and be guided by a detailed manual. We formed an Intervention Working Group, led by Dr. Gwadz, the PI of the HTH study and Co-PI of the present study (with Dr. Linda Collins). The Intervention Working Group was made up of senior research scientists expert in AABH-PLWH, members of the target population, and experienced clinical interventionists, who applied these criteria in an iterative process using Intervention Mapping, to select the most promising components.

### Description of intervention components to be tested

The Intervention Working Group identified five discrete intervention components for inclusion, as well as a preparatory Core intervention session to be conducted with all participants. Each component has two “levels” to be compared in the fractional factorial design: either yes/provided vs. no/not provided (Components A-D), or short version vs. long version (Component E). The five components selected for study are described below. The present study will be a definitive test of the efficacy of each component selected. Components will be guided by detailed manuals and will be culturally appropriate. Further, components will be individually tailored on substance use, mental health problems, and sexual minority status; manualized “algorithms” will be used to query or provide feedback (from baseline data) on these indices, followed by a series of prompts to guide the individually tailoring.

#### Core intervention session (~60 min)

All participants will receive a foundational Core intervention session. The goals of this component are to: 1) foster engagement and build trust/relationships and 2) provide standard treatment education on the current U.S. Department of Health and Human Services recommendations for frequency of HIV care appointments and timing of ART initiation [104, 105]. The primary theoretical target is HIV treatment knowledge.

#### Component A: MI counseling sessions, ~60–90 min each, 4 sessions

Sessions will be conducted with participants individually and made up of discrete exercises. Each session will include 1–2 culturally based video narrative segments to highlight key issues and foster discussion [106, 107]. Session 1 addresses barriers to HIV care. Sessions 2 and 3 target barriers to ART (S2: evoking barriers, fostering readiness; S3: decisions, plans). Session 4 addresses adherence, individual barriers and their solutions in depth, and finalizing care/ART plans. This component’s primary theoretical targets are health beliefs (e.g., outcome expectancies, self-efficacy, medical distrust), and emotions (e.g., concerns/fears of ART).

#### Component B: Pre-adherence preparation (2–6 wk. period)

The Health Resources and Services Administration (HRSA) provides guidelines for preparing PLWH-NECTA for treatment success [108–110], an approach supported by the research literature [69, 105, 110–112]. Component B is grounded in the HRSA guidelines. Its goals are to prepare the physical and social “adherence environment,” put long-term ART supports in place, and build adherence skills. Component B is flexible and individualized and will first entail an in-person orientation home session (< 90 min) to assess readiness for ART, identify individual barriers to adherence prior to initiating ART (e.g., substance use), link adherence to daily activities to build habits, put educational and visual aids and reminders in place, understand side effects, identify and involve long-term supports/supporters who can reinforce successes, and plans to minimize lapses if doses are missed. With the participant’s consent, the health care provider will be queried regarding the simplest dosing schedule [108, 113]. Next, a series of trial runs, with feedback, will be conducted (1–4 week-long trials). Trial runs will comprise 1-week practice trials with a daily pill regimen similar to the actual future ART regimen (obtained from providers, if possible) but using vitamins. Adherence to vitamins will be monitored with medication event monitoring system (MEMS) caps or a similar electronic adherence monitoring device, to help participants work toward a goal of >85% adherence [114]. After each week-long trial, participants will receive feedback from the study interventionist on their adherence patterns, a key strategy to boost motivation [84], and barriers of/facilitators to adherence, if any, will be explored. Participants will make a personal decision about ART initiation with their providers; those with <85% adherence will *not* be discouraged initiating ART. This component’s primary theoretical target is behavioral skill to manage ART adherence.

#### Component C: Peer mentorship (regular interactions with a highly trained “successful” peer mentor [4 months])

Linking PLWH with peer mentors is an efficacious approach to HIV-related behavior change [15, 115–121]. Successful peer mentors (i.e., demographically similar PLWH who have consistently engaged in care and are taking ART with good adherence) can serve as credible role models and challenge negative peer norms about HIV care and ART [15, 115, 118]. The training curriculum for and core elements of Component C are based on the HRSA-funded Peer Education & Evaluation Resource (PEER) model [122]. Meeting approximately weekly face-to-face or by phone, the role of the peer mentor will be to: provide informal counseling; model healthy HIV behavior; provide practical tips for managing care/ART based on *his/her personal experience*; and provide resources to address barriers to care/ART [122, 123]. This component’s primary theoretical targets are peer modeling and peer norms. Secondary theoretical targets are social support and stigma.

#### Component D: Focused support groups (6 groups, ~90 mins. Each, every 2–3 weeks over 4 months)

Support groups can address the social isolation and stigma endemic among PLWH-NECTA [124–131]. Component D aims to provide emotional and instrumental support, reduce stigma, give acceptance or validation, and encourage shifts in perspective [132, 133]. Groups will be guided by the MI approach, facilitated by a skilled interventionist, focus on barriers to and decisions regarding care/ART, provide general social support, and attend to issues MSM, substance users, and those with mental health concerns face [134]. This component’s primary theoretical targets are social support and stigma regarding care/ART status. This is the only intervention component where participants from the different experimental conditions will engage with each other, raising the possibility of contamination among participants. A description of possible types of contamination and procedures to prevent contamination are described below.

#### Component E: Navigation (3 months [short] vs. 6 months [long])

Navigation is an efficacious, flexible, individualized, strengths-based approach to assist PLWH in identifying and overcoming barriers to health services [135–139]. Participants will be randomized to receive a short (3 months) or long (6 months) period of navigation [34, 140]. All participants receive at least the short version of this component because of the primacy of structural barriers to HIV care and ART, and need for ancillary services among PLWH-NECTA (e.g., for substance use and mental health), although the optimal duration of navigation is not known [136, 140]. Component E is based on the HRSA HIV System Navigation model [136]; delivered by a trained interventionist; menu-based; and highly focused. Core elements include: an initial face-to-face meeting (< 90 mins.) for review of participant’s readiness for and barriers to care/ART, including substance use and mental health, and creation of a Change Plan/Action Plan, and a minimum of weekly phone (including text messages), email, and in-person meetings during the navigation period, depending on need. The menu of activities includes: screening and “Fast Track” referrals for substance use, mental health, and other problems including MSM-friendly sites; communication with primary care provider, as needed, about the participant’s service needs and care/ART plans; and accompaniment to health care appointments. This component’s primary theoretical target is ameliorating structural barriers to care and ART.

### Outcomes

Study outcomes will be assessed using objective data. The primary outcome is HIV virologic suppression analyzed as a dichotomous measure (assessed via lab report). Secondary outcomes include 1) absolute HIV VL (a continuous measure, assessed via lab report), 2) adherence to ART as assessed by ART concentrations in hair samples [103], and 3) engagement in HIV primary care, defined below (assessed via medical records) [105].

### Study setting

The study will be located in New York City, which has a large HIV epidemic, with approximately 115,000 PLWH, >75% African American/Black and Hispanic and ~55% MSM. Comparable to other urban areas, New York City has a large network of HIV care settings and all PLWH have access to care and ART [141]. Nonetheless, at the time the study was planned, New York City data indicated 45% were not retained in care, 49% were not taking ART, and 59% were not virally suppressed [142]. Thus >50,000 PLWH-NECTA reside in the local area, overwhelmingly African American/Black and Hispanic, concentrated in geographic areas with elevated rates of poverty [141, 143]. We will locate a project field site in one of the geographical areas with high rates of poverty and prevalent HIV (e.g., in central Brooklyn) and project activities will take place there.

### Trial design

The effects of the five individual components will be examined by means of an innovative, highly efficient fractional factorial experiment. A factorial experiment is an efficient way to examine these five components, for two reasons. First, factorial experiments separate component effects, enabling estimation of the main effect contribution of each candidate component and interactions between components. Second, factorial experiments can be economical compared to alternative designs, because they often require substantially fewer participants to achieve the same statistical power for component effects [36, 144].

As noted above, we plan to conduct a fractional factorial experiment involving five factors, each with two levels. The first four factors are: (A) MI counseling sessions; (B) Pre-adherence preparation; (C) Peer mentorship; and (D) Focused support groups. For components A-D, the levels of each of these factors are “no” (not included in the intervention) and “yes” (included in the intervention). The levels of the fifth factor, (E) Navigation, are “short duration” navigation (3 months) and “long duration” navigation (6 months).

**Fig. 2:**
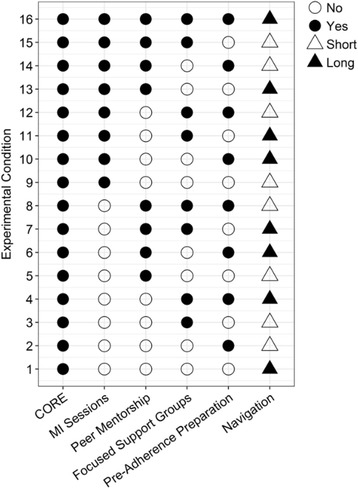
Conditions in the fractional factorial design

Our power analysis, presented below, indicates that *N* = 512 is sufficient to maintain power of at least 0.8. Conducting five individual experiments, one for each component, would require *N* = 2560, or five times as many participants as the factorial experiment, and comparative, dismantling, and constructive experimental designs would require *N* = 1536, or three times as many participants [36]. The fractional factorial design selected for this study requires 16 experimental conditions. The 16 conditions in the design selected for the present study are presented in Fig. 2, and procedures used to select these conditions are described below.

This design should not be considered a 16-arm RCT. The purpose and logical underpinnings of the factorial experiment, as well as the logic behind powering factorial experiments, are different from those of an RCT. The purpose of an RCT is direct comparison of the efficacy or effectiveness of two or more versions of an intervention. By contrast, although each of the 16 conditions in Fig. 2 represents a viable version of the enhanced HTH intervention, a factorial design *never* calls for direct comparison of these experimental conditions to see which one is best. Instead, the purpose of a factorial experiment in this context is to identify which *components* are (a) efficacious and/or (b) augment the efficacy of other components, so that we can select the ones that form the most cost-effective intervention. Efficiency comes from basing estimates of all estimated main effects and interactions on all 16 conditions in the factorial experiment. For example, the main effect of MI counseling sessions will be estimated by comparing the mean outcome across Conditions 1–8 vs. the mean outcome across Conditions 9–16. All participants are included in the estimate of each main effect. This is quite different from how RCTs are analyzed, and is why factorial experiments can have a relatively small per-condition sample size and still have excellent power if the total *N* is sufficient [36, 144]. The fractional factorial design does not contain a traditional control group; it does not require one, because individual conditions are never compared [36]. Instead, each factor has two levels, one of which serves as a control for that factor.

Other advantages of the factorial experiment include that cost-effectiveness can supplement efficacy as criteria for determining which components will be included in the final optimized intervention, thereby increasing the pre-test likelihood that the MOST-engineered intervention is cost-effective. If a component is efficacious but with a much higher cost than other components with comparable efficacy, the high-cost component can be excluded from the final intervention. In addition, the factorial experiment enables examination of mediators of individual intervention component effects, for a detailed look at how components operate. It also allows for the examination of and moderator effects. Regarding moderators, we will conduct exploratory analyses to examine whether gender, race/ethnicity, substance use patterns, sexual minority status, and other relevant variables are moderators of component efficacy. This will inform future research aimed at developing adaptive interventions [145] made of different combinations of components tailored to respond to individual differences (Aim 2).

### Explanation for choice of experimental conditions

A complete factorial experiment would have 2^5^ = 32 experimental conditions. To conserve resources and reduce logistical complexity, we have chosen an innovative 2^5–1^ *fractional factorial design* [146] that cuts the number of experimental conditions in half, to 16. A fractional factorial design is made up of a strategically selected subset of the experimental conditions required in a complete factorial design. These 16 conditions were selected based solely on statistical considerations [36]. We used PROC FACTEX in SAS to select the design presented in Fig. 2 [147]. These 16 conditions included in the fractional factorial design are based on prioritizing estimation of intervention component main effects and two-way interactions.

The tradeoff for the economy gained by using a fractional factorial design is that some effects become entangled or “aliased.” The fundamental principle underlying fractional factorial designs is to construct a study so the effects of primary interest are aliased with effects not expected to be large or important, typically higher-order interactions. In our design, each main effect is aliased with a four-way interaction, and each two-way interaction is aliased with a three-way interaction. Because our theoretical model (presented in Fig. 1) does not specify any sizeable three-way or four-way interactions, we find this aliasing of effects an acceptable price to pay for a dramatic reduction in research implementation costs.

### Recruitment

The sampling plan is based on a proven efficient strategy [33]. PLWH-NECTA, even those out of care, tend to be networked with other PLWH through HIV and general social service and substance use settings [7, 148–150], and through MSM social, drug use, and sexual networks [151, 152], although a minority are not networked [67]. The sampling plan, a hybrid recruitment strategy, is informed by literature on recruiting hard-to-reach populations, which calls for extended timeframes, appropriate resourcing costs, formative research, and community partnerships [153–155]. The sampling plan has three main elements: identification of diverse venues where PLWH-NECTA can be located by professional and peer experts, targeted sampling by staff/peer recruiter teams, and peer-to-peer recruitment. Specifically, a Community Advisory Board (CAB) comprised of local experts and “successful” members of the target population (former PLWH-NECTAs) will meet bi-monthly. This CAB will identify diverse recruitment venues. The hybrid sampling plan will entail regular targeted sampling events conducted by staff and former PLWH-NECTA from these organizations. Peer-to-peer recruitment [106] will begin with a small number of “initial seeds” (*N* = 5–15) drawn from the targeted sampling venues and the CAB. Seeds will be given 3–8 coded recruitment coupons and will be asked to recruit peers (whom they know by name or face, are living with HIV, and they believe/suspect are not engaged in care and/or on ART) for which they will receive modest compensation ($10/peer) [106]. Peers will be screened for eligibility and then have the opportunity to recruit other peers until sample size goals are met. Sampling will take place in study months 7 to 33 (27 months, 19 participants/month).

### Eligibility criteria

Eligibility criteria include: 1) age 18–65 years; 2) African American/Black or Hispanic race/ethnicity; 3) HIV diagnosed for at least 6 months (HIV status confirmed with medical documentation); 4) has not taken ART in the past 6 weeks (the period of time assessed by a hair assay, described below, and a reasonable period of time not on ART for the present study); 5) sub-optimal engagement in HIV care (assessed from the medical record, defined as less than 1 visit in every 4-month period in the past year [two of them at least 90 days apart], pro-rated for those diagnosed less than a year ago) or ≥2 missed visits (without prior cancellation) in the past year [156]; 6) reside in the New York City metropolitan area; 7) not planning to leave the New York City metropolitan area in next year; 8) not actively psychotic based on screening instrument [157]; 9) not a participant in the preliminary pilot HTH study; 10) able to conduct research activities in English or Spanish; 11) willing to provide hair sample (if possible), blood samples (to assess CD4, VL), and a Medical Report Form ([MRF], described below, to assess health care attendance); 12) willing to participate in a Core intervention session and be randomly assigned to 1–5 intervention components.

### Participant timeline

**Fig. 3:**
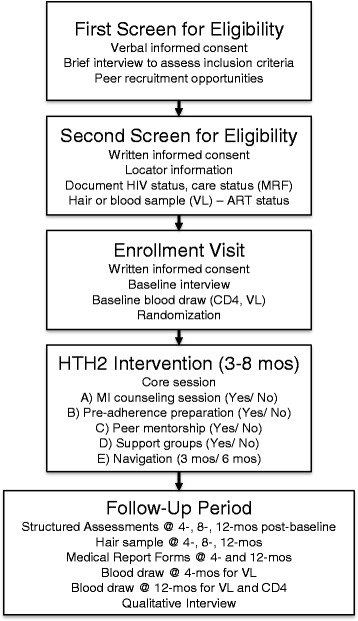
Sequence of HTH2-MOST study activities

An easy-access two-step screening procedure has been designed for efficiency and ease of completion, while fostering engagement and trust (Fig. 3).

#### Step 1. First screening interview (by phone) for eligibility

Verbal consent will be obtained and a structured pre-screening interview will be conducted to preliminarily screen for eligibility (criteria assessed by self-report). If preliminarily eligible, next steps to determine eligibility will be explained.

#### Step 2. Second screening interview for eligibility (in person)

Written informed consent for the remaining screening procedures will be obtained, as well as locator information. HIV status will be confirmed with medical documentation provided by the participant, then a hair sample collected to test whether the participant has used ART in the past 6 weeks, and a signed Release Form for Medical Records Office and Health Insurance Portability and Accountability Act (HIPAA) authorization form for the MRF will be obtained. Staff will outreach to the Medical Records office to obtain information on attendance at medical appointments. When MRF and hair results are received (~2–3 weeks), study eligibility will be determined.

#### Screening contingency plans

Those who cannot provide documentation of HIV status (~25%) will receive pre-test counseling and a point-of-care HIV test. Further, in past research ~30% of PLWH-NECTA could not provide a MRF because they did not have a regular health care provider [34]. In such cases, self-reported care engagement information will be accepted. If a hair sample cannot be obtained, a blood specimen will be obtained and HIV VL ≥ 1000 copies/mL will serve as a reasonable proxy for ART status (i.e., not taking ART).

#### Step 3: The enrollment visit

This visit will entail written informed consent for remaining study activities, administering the baseline interview, obtaining a blood specimen for baseline CD4 and VL levels, randomizing the participant to an experimental condition, and scheduling the Core intervention session. Random assignment will be stratified by age (younger PLWH [18–35 years] vs. older PLWH [36–65 years]). The measures that comprise the structured baseline assessment are presented in Table 1.

**Table 1:** Assessment instruments

PROXIMAL MEDIATORS (to assess each intervention component)	
*Health beliefs (*i.e.*, outcome expectancies, care/ART necessity, distrust) and emotions (*i.e.*, fear)*	Outcome expectancies re: care and ART (9 items each; α = .93) [199]
	Care and ART Necessity scale (10 items each; α = .80) [200]
	HIV and ART distrust (10 items; α = .84); HIV health care provider distrust (11 items; α = .88); General medical distrust (7 items; α = .72) [201–203]
	Care & ART Concerns & Fears subscale (disclosure, side effects; 13 items; α = .80) [56, 200]
*Adherence behavioral skills*	Mean % adherence rating from up to 4 one-week trial periods via MEMS caps; HIV Medication Readiness Scale (10 items; α = .90) [204]
*Peer models and peer norms regarding HIV care and ART*	Peer models (number and quality of “successful” HIV+ peers in care, on ART; α = .90) [205]
	Subjective peer norms for HIV care and ART (6 items each; α = .84) [206]
*Social support and stigma associated with care, ART*	Social support (α = .88) [207]
	Stigma associated with taking or not taking HIV care and ART (3 items each; α = .73) [208]
*Structural barriers to care/ART*	HIV-related structural/ practical barriers to care, ART (α = .72) [136]
DISTAL MEDIATORS	Motivation and readiness for care and ART [209]
	Schedule of HIV appointments [210]
	ART Prescription [210]
	Ancillary treatment [211]
	Substance use frequency [212]
	Depression [213]
MODERATORS	Socio-demographic characteristics (age, biological sex, sexual minority status, race/ethnicity)
	HIV history and ART history
	Substance use [212]
	Depression [213]
	Anxiety
OTHER DESCRIPTIVE AND BACKGROUND VARIABLES	Housing status, transgender gender identity, employment status, health status; where receives HIV care, incarceration; sex work history; reasons not on ART or discontinued ART; ART side effects (at FU); HIV treatment knowledge [214]; Methadone Maintenance/opioid substitution therapy; satisfaction with HIV care [215]

### Sequence of intervention components

Some of the 16 conditions are intensive but delivery is feasible, based on our experience with complex interventions. As Fig. 2 shows, the majority of conditions have 3–4 components. Sequences of components will follow pre-established rules: the Core intervention is delivered first, MI counseling sessions (where assigned) will come second, components may be provided simultaneously in some cases but will be scheduled so they do not conflict, and pre-adherence preparation will be scheduled to start after a minimum of 1.5 months of navigation. All participants receive the core intervention and 3 or 6 months of navigation, with the intervention periods ranging from ~3.25 to ~8 months. Participants receive modest compensation for intervention activities (e.g., $25 for a session, group, or other activity plus funds for two-way public transportation).

### Preventing contamination across experimental conditions

There are two main forms of contamination that could arise in the present study if a participant learns what other components (and other forms of intervention/treatment) a fellow participant is receiving. One potential form of contamination would be “resentful demoralization;” that is, participants feeling disappointed or disgruntled by their treatment in the study relative to other participants, which could then possibly reduce their motivation to engage in the study [158]. A second concern would be that a participant would be triggered to pursue similar types of activities outside of the study to compensate for what is not being received in the study. There are two main places that contamination could occur: in study waiting areas, and in the focused support group component, when participants from different experimental conditions come together. To prevent contamination from either the “waiting room” or support group component, participants will be informed at enrollment that study involvement and compensation varies across participants, in order to manage expectations. Further, at enrollment we will ask that participants not discuss the specifics of study components with other participants. Then, within the context of the focus support groups, the facilitator will attend to and discourage discussion of other components by participants in the groups. We may not be able to eliminate contamination entirely, but we can takes steps to minimize it.

### Blinding of staff members to intervention content

To foster fidelity to the intervention manuals and maintain the integrity of each separate component, interventionists will each deliver only one type of component and will not be trained in the specific content of other components. For example, interventionists trained to provide navigation will not be trained in the specifics of the other four components, and will not deliver any other component. Further, staff will be blind to participants’ intervention arm assignments where possible.

### Intervention quality assurance

We will establish and maintain treatment fidelity to the 16 conditions and the core elements of each component. A Research Electronic Data Capture (REDCap) database will be programmed to reflect the participant’s intervention assignments and will prompt interventionist action steps. REDCap is a secure web application for building and managing online surveys and databases. After each contact, interventionists will complete fidelity checklists. Audiotaped sessions will be randomly selected and rated for treatment fidelity by independent raters using the MI Treatment Integrity (MITI) coding system or a similar coding system. A clinical supervisor will review recordings of group sessions. Interventionist fidelity will be reviewed in bi-monthly individual supervision meetings.

### Sample size

A total of 512 participants will be enrolled in the experiment. For the primary outcome, HIV viral suppression at the final follow-up, we used PASS [159] to estimate the sample size needed for individual main effects of intervention components corresponding to odds ratios (OR) of 1.9 in logistic regression, given α = .05. A transition from viremia to viral suppression has clear clinical significance for individual patients, and the effect size reflects the need to have at least a moderate impact on the rate of suppression for public health impact. Assuming participants not receiving or receiving the lowest intensity of each component have a 20% chance of viral suppression at the final follow-up, a sample size of 404 provides 80% power to detect an OR of 1.9. To account for attrition of up to 20% of enrolled participants, we propose a total sample size of 512 participants to ensure complete data for at least 404. Given the proposed sample size, when the main effect of an intervention component on a continuous measure of a secondary outcome (e.g., log_10_ VL) or mediator is estimated in a linear model or independent-samples t-test, the sample size provides 80% power to detect a small standardized mean difference (*d* = .28). Moderator effects corresponding to an odds ratio of OR = 1 in one subgroup and OR = 3 in another can be detected with 76% if subgroups sizes are roughly equal.

### Randomization and data management

A secure, web-based, password-protected database built on a REDCap platform will be used to manage recruitment, eligibility assessment, randomization to the 16 experimental conditions, scheduling and tracking, baseline and follow-up assessments, and delivery of the intervention components (with cues, prompts, pull-down menus, Likert scales, and open ended responses).

### Collection of HIV care patterns using the medical report form (MRF)

We will obtain a MRF, a type of participant-facilitated chart review, at screening and the 4- and 12-month follow-up assessments, by contacting the Medical Records Office or health care provider where the participant receives HIV primary care, or asking participants to have their providers complete a MRF. The MRF will be completed by the provider and faxed to us in a secure fax line in a locked office at the New York University Meyers College of Nursing. The MRF is very brief (solicits the number of missed and kept HIV care appointments), so as to not burden health care providers and facilities. In the event these data cannot be obtained for a participant, for example, because the participant does not have a primary care provider, such data on health care attendance patterns will be collected by self-report.

### Assessing ART adherence levels in hair

Measuring ART exposure via hair is an objective and innovative biomarker of adherence. Average adherence to boosted protease inhibitors (PIs) is a better predictor of virologic suppression than duration or frequency of missed doses [160]. Further, hair levels of ART have been found to be stronger predictors of treatment outcomes than self-reported adherence [103, 161] or single plasma ART concentrations [161]. Dr. Monica Gandhi, a study collaborator, has developed methods to analyze protease inhibitors, Non-Nucleoside Reverse Transcriptase Inhibitors (NNRTIs), tenofovir (TFV), and emtricitabine (FTC) using liquid chromatography/tandem mass spectrometry (LC/MS/MS) [103, 162–166]. PIs and NNRTIs require 20–30 strands of human hair (~1–3 mg [mg]) and TFV or FTC from 50 to 100 strands of hair (~5–10 mg). These methods have been validated with good linearity (R^2^ > 0.99) and reproducibility (coefficient of variation [CV] < 15%) for all ART drugs. Moreover, many of the hair assays developed in our collaborating laboratory led by Dr. Gandhi have been peer-reviewed and approved by the NIAID Division of AIDS Clinical Pharmacology and Quality Assurance (CPQA) program [167, 168].

Hair collection is noninvasive and does not require specific skills, sterile equipment, or specialized storage conditions, and high rates of acceptability and feasibility of collecting hair samples for hair ART monitoring have been found in the Women’s Interagency HIV study (WIHS) [161, 169, 170]. In the present study, 100 strands of hair will be collected and assayed for TFV concentrations [163] in those on TFV-based regimens (a commonly-used agent in current regimens) [105]. For those not on TFV-based regimens, hair samples will be screened for the anchor antiretroviral (e.g. NNRTI, PI or integrase inhibitor). At follow-up, participants’ specific ART regimen will be logged from pill bottles or prescriptions, and hair analyses will be conducted for the relevant agents.

### Follow-up assessment schedule and activities

The follow-up (FU) periods and assessment schedule (4-, 8, and 12- months post-baseline) are based on the hypothesized timing and rate of change [171]. The FU schedule will allow assessment of the initiation and continuation of and adherence to ART, viral suppression, and patterns of engagement in care over time. Each FU includes a brief structured assessment battery (< 60 mins.); the 4-month FU also includes a blood draw (for VL), hair sample collection (if taking ART), and completion of a MRF (for assessment of HIV primary care visits) from participants’ HIV care site; the 8-month FU includes hair sample collection; and the 12-month FU includes a blood draw (CD4, VL), hair sample collection, and MRF. Specific reliable/valid assessment instruments for each mediator are presented in Table 1, as well as to assess socio-demographic and background characteristics. Time, resources, and cost of delivering each intervention component will be collected using forms created by the Drug Abuse Treatment Cost Analysis Program [172]. Participants receive modest compensation for assessments ($25), providing hair samples ($10), and blood specimens ($20), plus funds for two-way public transportation.

### Qualitative interviews and data integration

To add context and richness to our understanding of participants’ experiences with intervention components, advance understanding of barriers to care/ART, and inform future research, we will embed qualitative interviews into the study. A subset of participants will be purposively selected for maximum variation for qualitative interviews [173]. We will enroll *N* = 40 total, or until saturation on core constructs is reached [174]. Interviews will follow a semi-structured guide with a “start list” of key questions drawn from the theoretical model domains, and also allow for exploration of unanticipated themes. The use of the start list fosters data integration across qualitative and quantitative data sets, because the same core constructs are assessed in each. Analyses will be conducted by two qualitative researchers using Dedoose (a platform for mixed methods analysis). Participants receive modest compensation for the qualitative interview ($25), plus funds for two-way public transportation.

### Statistical methods

Intent-to-treat analysis will be our primary analytic approach and exploratory analyses will examine complier average effects of intervention components [175, 176]. Approaches to missing data will include full information maximum likelihood estimation [177] and multiple imputation [173]. In sensitivity analysis, missing data will be treated as failure to achieve the desired outcome. If data are missing not at random (MNAR), we will employ sensitivity analysis, using selection [107] or pattern mixture [178, 179] models.

#### Aim 1

Identify which of five components contribute meaningfully to improvement in the primary outcome, HIV viral suppression, as well as, absolute HIV viral load; ART adherence levels; and engagement in HIV care.

The primary outcome for Aim 1 is viral suppression at the final follow-up point (12-months post-baseline). Logistic regression will be used to estimate effects of components on the odds of viral suppression. Experimental factors will be effect coded to estimate main effects and two-way interactions of all five intervention components. The coefficient for a main effect term, multiplied by two and exponentiated, will estimate the effect of the component on the odds of viral suppression. Similarly, the coefficient for an interaction term, multiplied by two and exponentiated, will estimate interaction effects between intervention components on the odds of viral suppression. Similar logistic regression analyses will estimate effects of components on secondary outcomes. Linear regression will estimate effects of components on VL (after log_10_ transformation) and ART concentration in hair samples.

#### Relationships among participants

The sampling and intervention design may create clusters of participants whose outcomes are not fully independent. Participants with recruitment relationships may have more similar outcomes than two randomly selected participants. Also, participants receiving an intervention activity together may have more similar outcomes than randomly selected participants. Intraclass correlations or median ORs [180] will be estimated, and the impact of design effects on inferences will be considered.

#### Aim 2

Identify mediators and moderators of the efficacy of each intervention component.

Generalized linear model analysis will determine impacts of intervention components on mediators. MacKinnon and Dwyer [181] and MacKinnon [182] discuss how mediated effects can be calculated when the outcome or mediator variable is categorical. Probit regression, used to estimate indirect effects, will determine which mediators are related to viral suppression, after controlling for intervention components received. Intervention components may not be equally effective for all participants. The following factors, and others, may modify the relation between the intervention and outcomes: age, gender, sexual minority status, and substance use. The examination of potential moderator effects will involve forming interaction terms using the procedures described by Aiken [183] and Jaccard [184] and estimating simple effects. MOST enables estimation of moderator effects for each intervention component and component two-way interactions. Substance use will be thoroughly characterized in structured assessments using mainly measures approved by National Institute on Drug Abuse (NIDA) for the “Seek, Test, Treat, and Retain” initiative data harmonization effort. Given past research, we anticipate most participants (~80%) will have lifetime drug use and approximately half will have recent substance use. Importantly, we anticipate variation in a number of salient aspects of substance use among substance users (e.g., quantity and frequency of use, consequences of use, duration of use) will allow us to consider important intervention effect moderators. Identified moderators will be used to inform future development of adaptive interventions [145].

#### Aim 3

a) Using significance tests and effect size estimates obtained in Aim 1 analysis, identify components with efficacy, taking interactions into account; b) use modeling to estimate cost-effectiveness of possible packages composed of efficacious components; and c) identify the most cost-effective package.

The selection of the combination of intervention components that will make up the new multi-component “optimized” intervention will proceed as follows [35–37]. First, based on the experimental results, ineffective components will be eliminated. Components empirically demonstrated to be efficacious, and therefore candidates for inclusion in the optimized intervention, will be identified using procedures outlined in Collins et al. [37]. An initially selected component may be deselected if it interacts with another component in such a way as to undermine its effect, or a component not initially selected may be selected if it interacts with another component to enhance its effect. Then, drawing from the remaining components, the set of components/component levels that meets the optimization criterion, in this case cost-effectiveness, will be selected. Starting with effect sizes and costs of efficacious components, computer simulation methods will identify intervention packages that most increase population health for the magnitude of resources they consume (i.e., on the efficiency frontier of the cost-effectiveness plane). Enhancing our validated HIV simulation with new “states” (e.g., disengaged, engaged/not on ART, engaged/on ART but not adherent), we will consider downstream as well as immediate costs, and follow guidelines of the Panel on Cost-Effectiveness in Health in Medicine [185]. *Utilities* (preference-weighted quality-of-life measures used in cost-effectiveness analyses) will vary by CD4 count, and will be based on those used in the modeling analyses [186–191].

#### Uncertainty and sensitivity analyses

We will perform a probabilistic sensitivity analysis in which all inputs are simultaneously varied across their plausible ranges, and assess the proportion of runs that an intervention strategy remains on the efficient frontier. We also will perform a sensitivity analysis by strength of evidence [192, 193], where we vary an evidence “filter” that only allows data sources to inform input assumptions if they pass through the “filter” and meet the minimum standard of evidence, thereby assessing the lowest level of evidence filter compatible with a particular intervention strategy remaining on the efficient frontier.

#### Assumptions

We will make conservative assumptions about *duration of effects*, assuming they last only as long as the last observed follow-up, but will explore more optimistic assumptions in sensitivity analyses. We will base resource utilization not only on the costs of the intervention package itself, but also considering changes in attributable *downstream costs* (e.g. people re-linked to care might incur lower hospitalization expenses in the long-term because they maintain higher CD4 counts and are less likely to get AIDS). Relative trajectories of utilization pathways (drug costs, outpatient costs including labs and visits, and inpatient costs) with versus without re-engagement in care will be estimated based on our simulation. We will perform analyses from different *perspectives* (societal and payer), *time horizons* (infinite, 20, 10, and 5-year), and *discount rates* (5%, 3%, and 0%) but with base case assumptions in accord with established guidelines [186, 194–198].

### Data monitoring

We will perform reliability checks on measures at an interim analysis point. Construct validity of key measures will be assessed using measurement models within a structural equations format (using Mplus).

### Fidelity, process ratings, and quality assurance

As noted above, after each intervention session/navigation contact the interventionist will complete process ratings. These ratings will be used in regular supervision sessions to insure fidelity to the intervention manual. Sessions will be audiorecorded (if participants give their signed informed consent) and ~10% of the tapes selected at random will be reviewed for quality assurance and supervision purposes by an independent rater who will complete a standard process rating checklist. They will be reviewed within approximately a month of their taping to ensure timely feedback and then destroyed. The facilitators will attend monthly supervision meetings with a senior clinician where quality assurance, clinical issues, and intervention fidelity issues will be reviewed. The study will employ a number of procedures to address “drift” from intervention fidelity including on-going supervision meetings with facilitators and senior staff, regular monitoring of process ratings, and “booster” training of facilitators based on the intervention manual provided as needed.

### Check on level of missing data and any patterns by item, data source, or staff person

We propose to use the SPSS Missing Values Analysis (MVA) program to identify possible non-random patterns of missing data. When items, data sources, or staff are associated with more than 10% missing data that are not due to planned interview skip patterns, we will determine the causes of missing data and implement strategies to reduce it (e.g., retraining of staff).

### Harms

The study will make use of a Data Safety and Monitoring Board (DSMB). Several mechanisms will be put in place to monitor potentially adverse events that participants may experience while enrolled in the study, whether they are related to project participation or not. These events are classified as either Reportable, Adverse, or Not Harmful/Expectable, as described below, and will be reported to the New York University (NYU) and Pennsylvania State University Institutional Review Boards (IRBs), DSMB, and the sponsor’s Program Officer accordingly, as described below. Social harms will be assessed with a structured instrument at each FU point, and social harms may be reported during intervention activities. A Reportable Event is an unanticipated problem involving risks to participants or others (“Unanticipated Problem”) and any event or information that (1) was unforeseen and (2) indicates that the research procedures caused harm to participants or others or indicates that participants or others are at increased risk of harm.

### Research ethics approval

The study protocol will be approved by the IRB of the New York University School of Medicine (the IRB of record), Pennsylvania State University (Dr. Linda Collins, Co-Principal Investigator), and Binghamton University (Dr. Leo Wilton, Co-Investigator).

### Consent

Verbal consent will be obtained and a structured pre-screening interview will be conducted to preliminarily screen for eligibility (criteria assessed by self-report). Signed informed consent for the remaining screening procedures will be obtained. Those found eligible will provide signed informed consent to enroll in the study. Participants will provide separate signed consent to have the qualitative interviews and intervention sessions audio-recorded. Participants may decline to have their qualitative interviews or intervention sessions recorded and still continue with the interviews or sessions. The voluntary nature of all study activities is emphasized in the consent forms. The participant will be provided a copy of the consent form that includes contact information for the research team members and the NYU IRB. Participants can use this contact information to report adverse events or unanticipated problems.

### Confidentiality

All participants will receive a Participant Identification Number (PID) that will be used for all interviews, forms, materials, hair samples, blood specimens, transcripts, and intervention materials. No other information that would disclose the participant’s identity will be found on any interview or form. Paper forms will be kept without serostatus identification in locked cabinets at NYU. Only the consent form, locator form and a Master Participant Log File will link the participant’s name to the identification number. Staff receives training about confidentiality and the New York State HIV Confidentiality Law. Participants will provide verbal informed consent for the brief screening interview, and for those found preliminarily eligible, signed informed consent for remaining study activities (assessments, blood specimens, hair samples, intervention, peer recruitment).

## Discussion

The goal of elimination of HIV transmission in the United States will not be achieved without improvements in engagement along the HIV care continuum. The present study targets the large population of PLWH in the United States who are both insufficiently engaged in HIV primary care and not taking ART, who are mainly African American/Black and Hispanic. The National Institutes of Health has emphasized the urgent need for new research approaches to advance intervention science, and the proposed project employs a new, potent, and innovative research methodology, the multiphase optimization strategy (MOST), a framework for developing highly efficacious, efficient, scalable, and cost-effective interventions. The proposed study has the highest public health significance: it addresses a vulnerable population of PLWH, including the critically important subpopulations of MSM and substance users; will develop an efficient and cost-effective intervention to increase engagement along the HIV care continuum for these vulnerable groups; and addresses two research priorities areas from the National Institutes of Health Office of AIDS Research (NOT-OD-15-137), namely, engaging PLWH in prevention/treatment services, and reducing HIV/AIDS-related racial/ethnic disparities.

## Abbreviations

AABH-PLWH: African American/Black and Hispanic persons living with HIV
ART: Antiretroviral therapy
CAB: Community Advisory Board
CPQA: Clinical Pharmacology and Quality Assurance
CV: Coefficient of variation
DSMB: Data Safety and Monitoring Board
FTC: Emtricitabine
FU: Follow-up
HIPAA: Health Insurance Portability and Accountability Act
HRSA: Human Resources and Services Administration
HTH: Heart to Heart Study
IRB: Institutional Review Board
LC/MS/MS: Liquid Chromatography/Tandem Mass Spectrometry
MEMS: Medication Event Monitoring System
Mg: Milligrams
MI: Motivational Interviewing
MITI: Motivational Interviewing Treatment Integrity
MNAR: Missing not at random
MOST: Multiphase optimization strategy
MRF: Medical Report Form
MSM: Men who have sex with men
MVA: Missing Values Analysis
NIDA: National Institute on Drug Abuse
NNRTI: Non-Nucleoside Reverse Transcriptase Inhibitor
NYU: New York University
OR: Odds ratio
PEER: Peer Education & Evaluation Resource
PI: Protease inhibitor
PID: Participant identification number
PLWH: Persons living with HIV
PLWH-NECTA: Persons living with HIV - not well engaged in care nor taking antiretroviral therapy
PWID: Persons who inject drugs
RCT: Randomized controlled trial
TFV: Tenofovir
VL: Viral Load
WIHS: Women’s Interagency HIV study
